# Dual-Energy X-Ray Absorptiometry Compared to Computed Tomography for Visceral Adiposity Assessment Among Gastrointestinal and Pancreatic Cancer Survivors

**DOI:** 10.1038/s41598-019-48027-1

**Published:** 2019-08-08

**Authors:** Adriana M. Coletta, Ann H. Klopp, David Fogelman, Aaroh M. Parikh, Yisheng Li, Naveen Garg, Karen Basen-Engquist

**Affiliations:** 1 University of Texas MD Anderson Cancer Center, Department of Behavioral Science, Houston, TX USA; 2 Huntsman Cancer Institute, Cancer Control and Population Sciences Program, Salt Lake City, UT USA; 3 University of Utah, Department of Health, Kinesiology and Recreation, Salt Lake City, UT USA; 4 University of Texas MD Anderson Cancer Center, Department of Radiation Oncology, Houston, TX USA; 5 University of Texas MD Anderson Cancer Center, Department of Gastrointestinal Medical Oncology, Houston, TX USA; 6 University of Texas MD Anderson Cancer Center, Department of Biostatistics, Houston, TX USA; 7 University of Texas MD Anderson Cancer Center, Department of Diagnostic Radiology, Houston, TX USA

**Keywords:** Cancer imaging, Pancreatic cancer, Cancer imaging, Gastrointestinal cancer

## Abstract

Dual-energy x-ray absorptiometry (DXA) for visceral adipose tissue (VAT) assessment is used as an alternative to computed tomography (CT) for research purposes in apparently healthy and clinical populations. It is unknown whether DXA is comparable to CT among cancer survivors, especially in cases where VAT assessment may be affected by treatment history and side effects and become more challenging to assess, such as a history of surgical gastrointestinal resection and/or ascites. The purpose of this study was to determine the level of agreement between DXA and CT when assessing VAT area and volume among cancer survivors. One hundred Gastrointestinal and pancreatic cancer survivors underwent abdominal and pelvis CT and whole-body DXA within 48 hours. Bland-Altman analysis revealed that in women and men, DXA VAT-area estimates were larger and smaller, respectively, and was consistently smaller in estimates for VAT-volume. Correlations from linear regression analysis revealed statistically significant positive correlations between measurement methods. Overall, while DXA VAT estimates are highly correlated with CT VAT estimates, DXA estimates show substantial bias which indicates the two methods are not interchangeable in this population. Further research is warranted with a larger, more homogeneous sample to develop better estimates of the bias.

## Introduction

Obesity is a disease of epidemic proportions, with nearly one-third of the adult population in the United States considered obese^1^. Obesity is associated with excessive accumulation of body fat, which is stored in two different locations, the visceral and subcutaneous areas^2^. When comparing sites, visceral adipose tissue (VAT) has been shown to be more metabolically active in comparison to subcutaneous adipose tissue^2,3^. Excessive accumulation of VAT is linked with metabolic dysfunction and subsequent increase in risk of developing a chronic disease, such as type 2 diabetes, coronary artery disease, hypertension, and even some types of cancer^2–4^. With regards to cancer, the link between obesity and risk of specific cancers is established^4–6^. Further, excess VAT is associated with increased risk of developing certain cancers^7,8^, notably breast^6,9^ and colorectal cancer^6,10–14^. A recent review conducted by Donohoe and colleagues^2^ proposes how the endocrine and paracrine mechanisms resulting from excessive VAT may facilitate the tumor micro-environment. Thus, assessment of VAT may serve as a useful tool to identify risk of poor health outcomes and cancer occurrence or recurrence.

There are several methods used to assess VAT, including both standard-dose and low-dose computed tomography (CT), and magnetic resonance imaging (Dixon)^15,16^. Currently, use of CT is the most common method to assess VAT area^17^. Yet administering CT scans to assess VAT beyond clinical purposes raises concerns, such as additional exposure to ionizing radiation and cost. Examples of cases in which CT would be administered for VAT assessment beyond clinical purposes include research purposes among individuals who would not otherwise undergo CT for clinical purposes, research purposes when the timing of CT does not align with the timing of surveillance scans for clinical purposes, and research purposes when the scan is beyond the time-frame of surveillance scans, such as in survivorship studies. Therefore, in these cases, it is important to identify an alternative method to assess VAT that provides less ionizing radiation compared to CT. Of late, new software to estimate both VAT area and volume with dual energy x-ray absorptiometry (DXA) has been developed^18^. Compared to CT scans, whole body DXA scans are less expensive and expose the patient to a much smaller dose of radiation. Depending on the site of examination, with CT scans the average effective dose of radiation in adults ranges from 2,000–16,000 µSv^19^ compared to 0.1–4.9 µSv with DXA^20^. The DXA software estimates VAT area from an automatically marked region of interest at L_4_-L_5_ and estimates VAT volume based on the VAT area estimation, slice thickness, and a general scaling factor^18^.

The most widely used method for assessing VAT with CT scan estimates VAT area from a single slice at L_4_-L_5_ and typically utilizes Slice-O-Matic software (TomoVision, Canada). We recently developed and validated a method for assessing VAT volume with CT imaging: the Volumetric Quantification of Visceral Adipose using CT^21^. In this method the user is able to manually define the region of interest (i.e.- from the dome of the liver to the tip of the femoral heads), then delineate between subcutaneous adipose tissue and VAT with elliptical contours for a series of continuous slices within the region of interest^21^. Since the new DXA software estimates both VAT area and volume, it would be useful to compare accuracy of DXA VAT area and volume estimates to CT VAT area and volume estimates.

To date, some investigations have compared DXA VAT estimates to CT VAT estimates^22–26^, however, these investigations have been conducted in apparently healthy individuals. It is unknown whether DXA VAT estimates are comparable to CT VAT estimates among cancer survivors, especially in cases where VAT assessment may be affected by treatment history and side effects and become more challenging to assess, such as a history of surgical gastrointestinal resection and/or ascites^27^. As yet no investigations have compared the DXA software estimates of VAT volume to our newly developed and validated method^21^ for estimating VAT volume with CT. Thus the primary aim of the present investigation was to determine both the agreement between DXA software estimates of VAT area and CT Slice-O-Matic software estimates of VAT area, and DXA VAT volume and CT VAT volume estimates via our newly developed and validated software, among cancer survivors.

## Results

### Participants

Participants were primarily Caucasian (89% total, 89% for women and men) and non-Hispanic (90% total, 87% women, 94% men). Additionally, the majority of patients were diagnosed with adenocarcinoma (76% total, 79% women, 71% men), at the site of the pancreas (46% total, 45% women, 46% men) or colon (20% total, 19% women, 22% men), at cancer stage 4 (54% total, 49% women, 59% men), with active disease status (79% total, 74% women, 85% men). Active disease status is defined as recurrence, initial treatment, ongoing chemotherapy, stable disease, pending surgery, disease progression, seeking new treatment recommendation. The average age of all 100 patients was 61.5 ± 12 years, and the average BMI was 28.5 ± 7.3 kg/m^2^ (46 were female). Ninety-nine of the 100 participants were included in the VAT area analysis (62 ± 12 years old, 28.5 ± 7.4 kg/m^2^); 53 were female (61 ± 13 years old, 28.3 ± 8.5 kg/m^2^) and 46 were male (62 ± 11 years old, 28.6 ± 5.9 kg/m^2^). Significant differences were observed between men and women for height (p < 0.001), body weight (p < 0.001), waist circumference (p < 0.001), waist to hip ratio (p < 0.001), DXA body fat percentage (p < 0.001), DXA VAT area (p = 0.043), and CT VAT area (p = 0.003). Eighty-eight of the 100 patients were included in the VAT volume analysis (61 ± 12 years old, 28.0 ± 6.1 kg/m^2^); 48 were female (60 ± 13 years old, 27.9 ± 6.6 kg/m^2^) and 40 were male (62.5 ± 11 years old, 28.1 ± 5.7 kg/m^2^). Significant differences between men and women were not observed for DXA VAT volume (p = 0.123) but were observed for CT VAT volume (p = 0.004). Differences between men and women were also observed for height (p < 0.001), body weight (p = 0.001), waist circumference (p = 0.001), waist to hip ratio (p < 0.001), DXA body fat percentage (p < 0.001), and CT VAT area (p = 0.02), but not DXA VAT area (p = 0.123) (Table 1).

**Table 1:** Patient Characteristics (n = 99).

Variable	Group	Mean ± SD	P-level
Age	Females	61 ± 13	0.58
	Males	62 ± 11	
	Total	62 ± 12	
Body Weight (kg)	Females	75.9 ± 24.0	<0.001
	Males	91.3 ± 22.0	
	Total	83.1 ± 24.3	
Height (cm)	Females	163.4 ± 6.4	<0.001
	Males	178.3 ± 8.0	
	Total	170.3 ± 10.3	
BMI (kg/m^2^)	Females	28.3 ± 8.5	0.87
	Males	28.6 ± 5.9	
	Total	28.5 ± 7.4	
DXA VAT area (cm^2^)	Females	117.1 ± 55.0	0.04
	Males	143.7 ± 73.7	
	Total	129.5 ± 65.4	
CT VAT area (cm^2^)	Females	106.8 ± 78.6	0.003
	Males	168.5 ± 120.9	
	Total	135.5 ± 104.6	
DXA VAT volume (cm^3^)*	Females	613.3 ± 296.4	0.123
	Males	725.7 ± 381.4	
	Total	664.4 ± 340.4	
CT VAT volume (cm^3^)*	Females	3209.3 ± 1934.8	0.004
	Males	4686.9 ± 2722.7	
	Total	3881.0 ± 2427.5	
DXA Body Fat Percentage	Females	39.8 ± 6.6	<0.001
	Males	30.2 ± 5.8	
	Total	35.3 ± 7.9	
Waist Circumference (cm)	Females	90.8 ± 14.8	<0.001
	Males	102.7 ± 13.6	
	Total	96.3 ± 15.4	
Hip Circumference (cm)	Females	106.6 ± 13.4	<0.001
	Males	105.8 ± 11.5	
	Total	106.3 ± 12.5	
Waist/Hip Ratio	Females	0.85 ± 0.07	<0.001
	Males	0.97 ± 0.07	
	Total	0.91 ± 0.09	

Data presented as mean ± standard deviation with alpha <0.05. Females (n = 53); Males (n = 46); *Females (n = 48), Males (n = 40), Total (n = 88); BMI = body mass index.

### DXA VAT area and CT VAT area

For the total sample, findings from the Bland-Altman analyses revealed a bias of 6.0 cm^2^ (95% confidence interval (CI) −4.8, 16.8) with limits of agreement of −99 to 111 cm^2^. DXA VAT area estimates were smaller than CT VAT area estimates by 6.0 cm^2^ or approximately 4.4% (calculation of bias converted to %: (CT VAT area measurement - DXA VAT area measurement)/CT VAT area). The linear regression analysis yielded a significant positive correlation estimate between CT VAT area and DXA VAT area (r = 0.902, p < 0.001) along with a coefficient of determination (r^2^) of 0.813. Additionally, the residual standard deviation estimate was 45.5 cm^2^.

**Figure 1:**
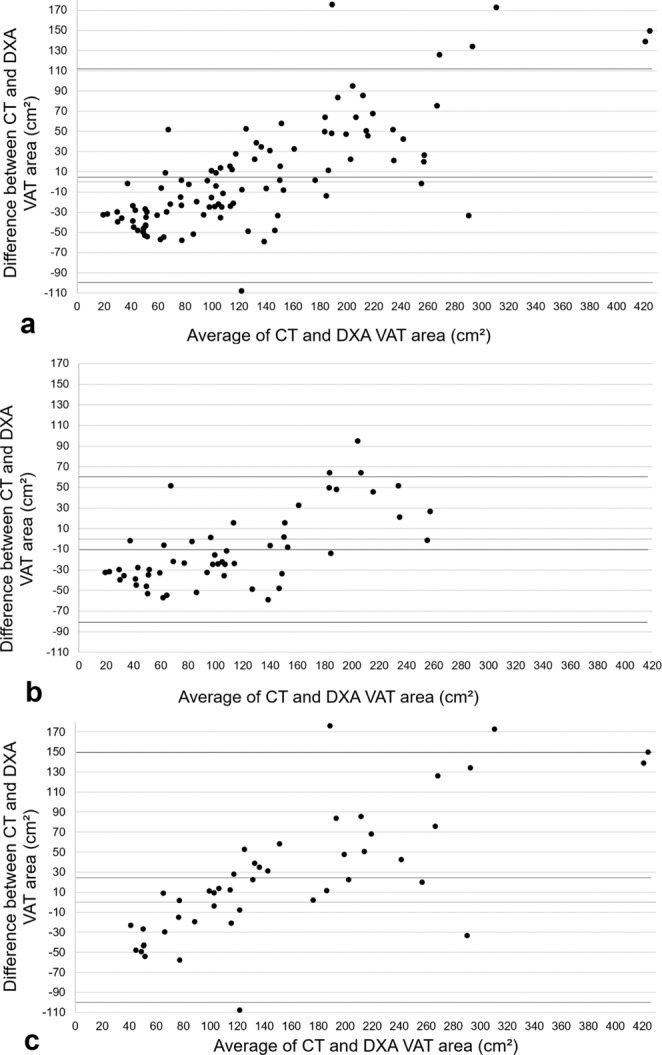
Agreement between methods for VAT area. Bland-Altman plots for: (**a**) Total sample (n = 99), (**b**) women (n = 56), (**c**) men (n = 43).

**Figure 2:**
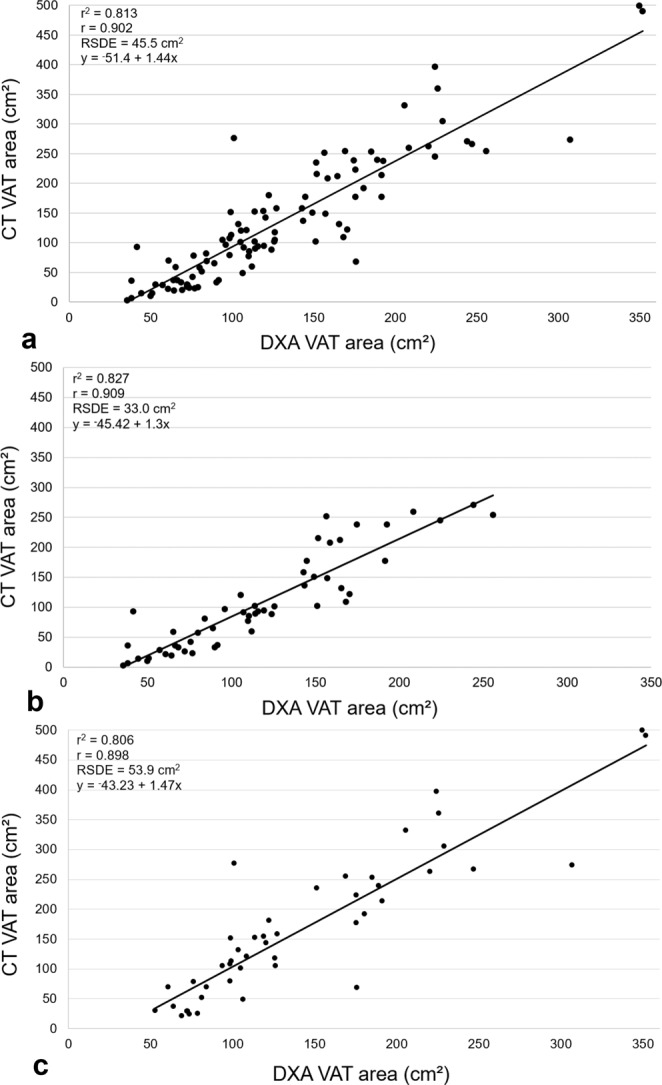
Correlations between methods for VAT area. Correlation plots for: (**a**) Total sample (n = 99), (**b**) women (n = 56), (**c**) men (n = 43); RSDE = residual standard deviation estimate.

When stratifying the sample by sex, Bland-Altman analyses revealed a bias of −10.3 cm^2^ (95%CI −20.4, −0.2) with limits of agreement of −82 to 62 cm^2^ for women, and a bias of 24.8 cm^2^ (95%CI 7.2, 42.5) with limits of agreement of −100 to 150 cm^2^ for men. That is, DXA VAT area estimates were larger compared to CT VAT area estimates by 10.3 cm^2^ (9.6%) for women and smaller than CT VAT area estimates by 24.83 cm^2^ (14.7%) for men. Significant positive correlations between methods were observed for women and men with r = 0.909 (r^2^ = 0.827, p < 0.001) and r = 0.898 (r^2^ = 0.806, p < 0.001) respectively. The residual standard deviation estimate from the regression analysis was 33.0 cm^2^ for women and 53.9 cm^2^ for men (Figs 1, 2).

### DXA VAT volume and CT VAT volume

Findings from the Bland-Altman analyses revealed a bias of 3,217 cm^3^ (95%CI 2,766; 3,667) with limits of agreement of −950 to 7,384 cm^3^ for the total sample. DXA estimate of VAT volume was smaller compared to CT VAT volume by 3,217 cm^3^ or approximately 83%. A significant positive correlation was observed between CT VAT volume and DXA VAT volume (r = 0.901, p < 0.001) along with a coefficient of determination of 0.812. Further, the residual standard deviation estimate from the regression analysis was 1,058 cm^3^.

**Figure 3:**
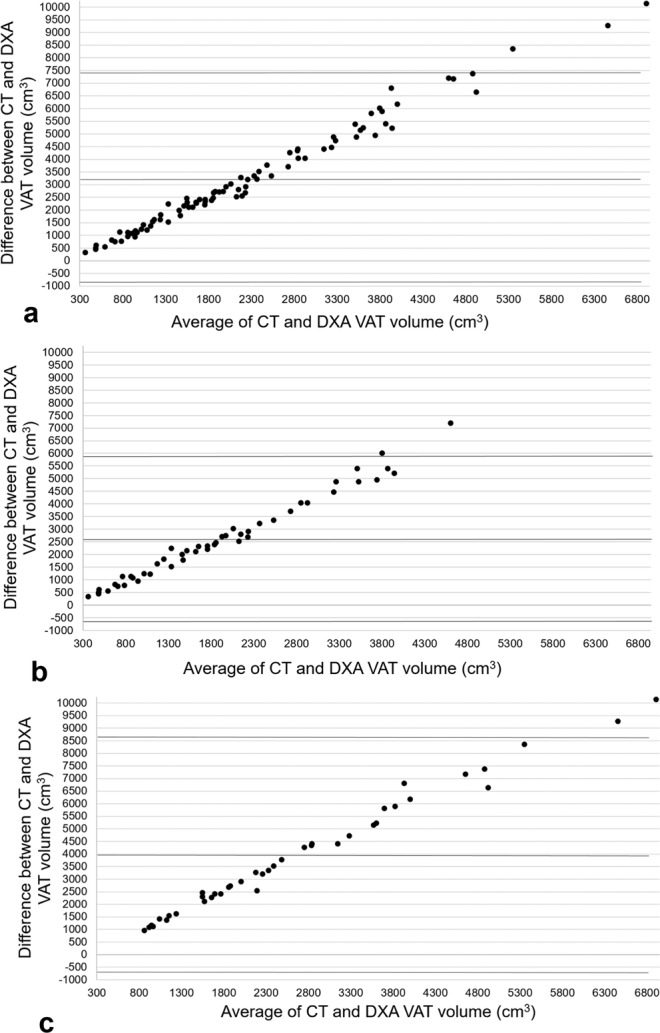
Agreement between methods for VAT volume. Bland-Altman plots for: (**a**) total sample (n = 88), (**b**) women (n = 48), (**c**) men (n = 40).

**Figure 4:**
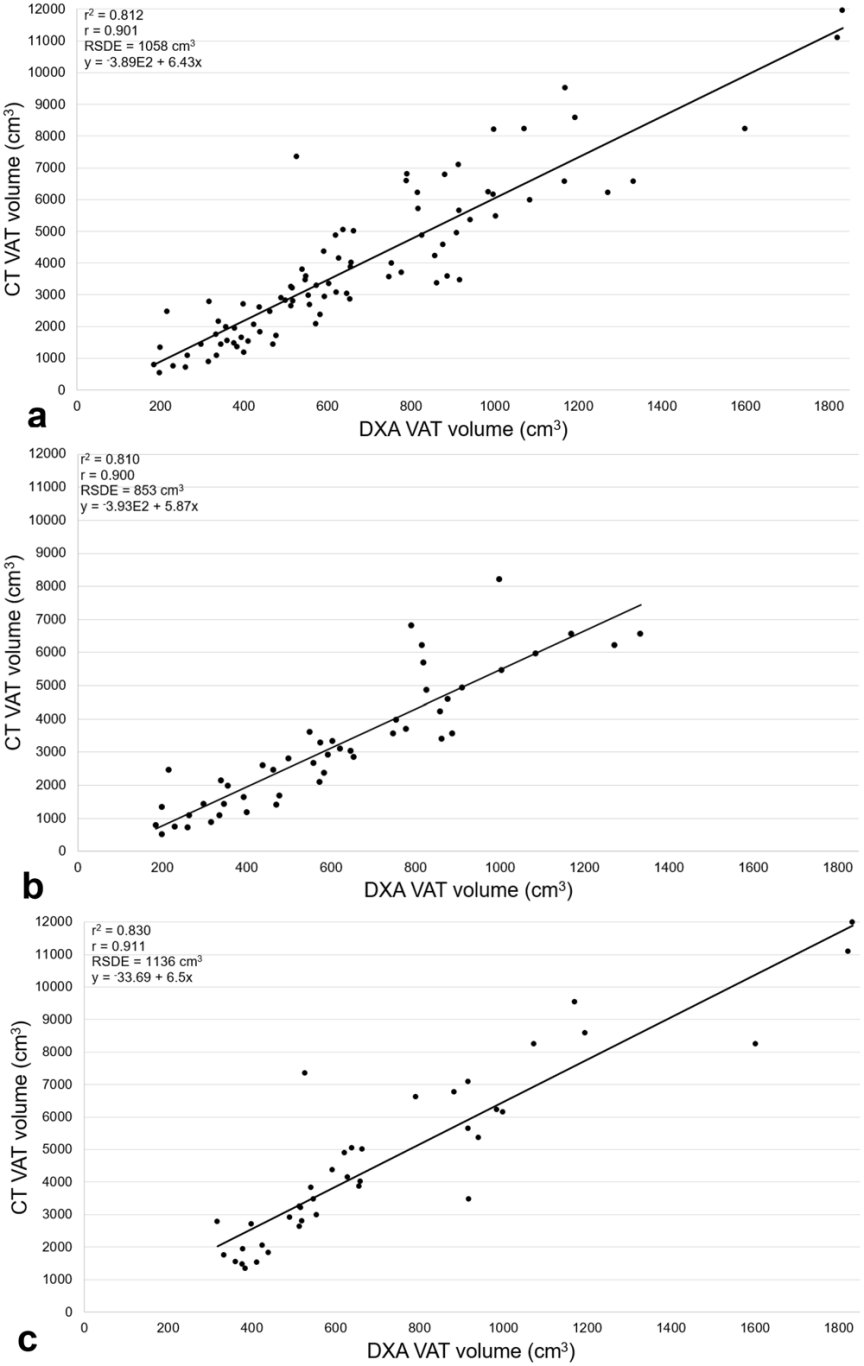
Correlations between methods for VAT volume. Correlation plots for: (**a**) Total sample (n = 88), (**b)** women (n = 48), (**c)** men (n = 40); RSDE = residual standard deviation estimate.

When stratifying the sample by sex, Bland-Altman analyses revealed a bias of 2,596 cm^3^ (95%CI 2,110; 3,082) with limits of agreement of −683 to 5,875 cm^3^ for women and a bias of 3,961 cm^3^ (95%CI 3,200; 4,722) with limits of agreement of −704 to 8,626 cm^3^ for men. That is, DXA estimate for VAT volume was smaller compared to CT VAT volume by 2,596 cm^3^ (81%) for women and 3,961 cm^3^ (85%) for men. Significant positive correlations between methods were observed for women and men with r = 0.898 (r^2^ = 0.806, p < 0.001) and r = 0.900 (r^2^ = 0.810, p < 0.001) respectively. The residual standard deviation estimate from the regression analysis was 853 cm^3^ for women and 1,136 cm^3^ (Figs 3, 4).

## Discussion

Previously, the use of DXA software to estimate visceral adiposity has been compared to CT scan in apparently healthy individuals^22–26^. The purpose of this study was to determine the agreement between DXA estimation of VAT area and volume and CT estimation of VAT area and volume among cancer survivors. We aimed to determine the utility of DXA to assess visceral adiposity by comparing DXA VAT area and VAT volume estimates to CT VAT area and VAT volume estimates.

For VAT area, we observed a difference of about 4.4% between methods for the total sample, with DXA VAT area estimates smaller than CT VAT area estimates by approximately 6.0 cm^2^. Additionally, we observed a strong positive correlation between measures (r = 0.90) with a relatively small residual standard deviation estimate of 45.5 cm^2^ from the regression analysis used for predicting CT scan measurement with DXA. Our relatively small residual standard deviation estimate indicates minimal variation of any observations that may be made from the linear regression equation. In comparison to a previous investigation conducted in apparently healthy women, a similar correlation coefficient was observed (r = 0.93), with a smaller residual standard deviation estimate (16 cm^2^)^23^. Interestingly, when stratifying our sample of gastrointestinal and pancreatic cancer patients and survivors by sex, we found a similar correlation coefficient (r = 0.91) and residual standard deviation estimate in women (23 cm^2^).

When stratified by sex, we observed a smaller percent difference and absolute value of the bias between methods in women (9.6%, −10.3 cm^2^) compared to men (14.7%, 24.8 cm^2^). Consistent with a previous investigation among apparently healthy adults that compared VAT mass assessed by DXA and MRI, similar results were found such that DXA VAT estimates were smaller in men and larger in women compared to MRI^28^. Collectively these findings suggest that regardless of measurement method, CT or MRI, DXA VAT area estimates have consistently been larger in women and smaller in men. In the present investigation, while DXA VAT area estimate appears to be more accurate (i.e.- smaller bias) in women compared to men, this observation may be attributed to lower measurement precision by the DXA machine among individuals with a higher mean VAT area, as evidenced by the higher mean VAT area in men and a greater degree of proportional bias observed within the Bland-Altman plot for men compared to both women and the total sample.

For VAT volume, we observed a large difference between methods for the total sample, about 83%, and by sex, 81% for women and 85% for men. DXA estimates of VAT volume were consistently smaller compared to CT. Bland-Altman plots for VAT volume revealed clear proportional bias between methods for the total sample and when stratified by sex. We attribute these findings to differences in the region of interest between methods, where the CT software consisted of a region of interest that expanded from the dome of the liver to the tips of the femoral heads and the DXA software region of interest was based on the VAT area measurement, slice thickness, and a general scaling factor^18^. Therefore, we are unable to dictate whether DXA estimates are larger or smaller compared to CT.

To our knowledge, there is only one other investigation^22^ that has compared agreement (Bland-Altman analysis) between DXA VAT volume estimation and CT VAT volume estimation. In contrast with our investigation, this investigation was conducted in an apparently healthy population. Findings contrast the present investigation such that the observed bias between DXA and CT VAT volume were not nearly as large (i.e.- total sample, n = 109: +567 cm^3^; females, n = 61: +67 cm^3^; males, n = 48: +43 cm^3^)^22^. Furthermore, the observed DXA VAT volume estimation was consistently larger than the CT VAT volume measurement among the total sample and by sex^22^. Again, differences in results between the previous investigation and present investigation may be attributed to variances in defining the region of interest for CT VAT volume estimation. The region of interest in the present investigation is much larger compared to the previous investigation^22^, 150 mm of the abdomen from S1.

This is the first study to assess agreement between methods for assessing VAT area and volume among cancer survivors, specifically gastrointestinal and pancreatic cancer survivors. We consider this a strength of the present investigation. Additionally, we consider use of our validated in-house software to assess VAT volume^21^ a strength of the present investigation, because this program allows the user to mark the specific region of interest for VAT volume estimation, ensuring adequate coverage of VAT throughout the entire abdominal region, and thereby providing a more accurate estimation of VAT volume. There are several limitations to the present investigation, which should be considered, such as use of a smaller sample size for comparison of agreement between methods, inclusion of patients with varying types and stages of cancers, inclusion of patients with varying degrees of response to treatment, and assessment of cardiovascular disease risk factors in relation to DXA and CT VAT estimates. Future research should consider a larger, more homogenous sample, consistency in region of interest utilized for VAT volume comparisons, and assessment of cardiovascular risk factors in relation to VAT estimates. Furthermore, the present investigation along with previous investigations that assessed agreement by conducting Bland-Altman analysis did not pre-set limits of agreements; thus considering findings from the present investigation and previous work, future work should include pre-set limits of agreement.

Taken together, DXA VAT area and volume estimates are highly correlated with estimates from CT scans among gastrointestinal and pancreatic cancer survivors. However, in terms of agreement, the DXA estimates show substantial bias which indicates the two methods are not interchangeable in this population. Further research is warranted with a larger and more homogeneous sample to develop better estimates of the bias. In addition, longitudinal research should be done to evaluate whether DXA can reliably estimate VAT changes over time in this population.

## Methods

### Recruitment and study protocol

**Figure 5:**
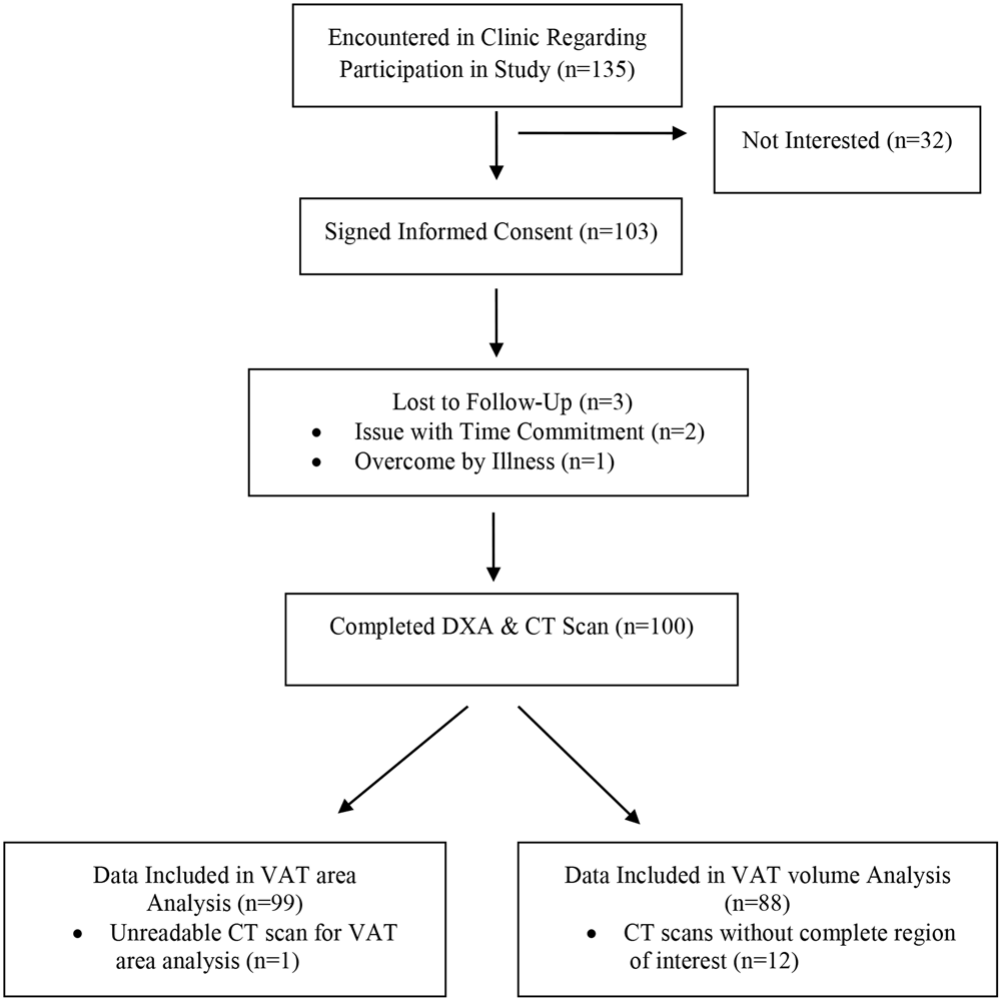
Consort Diagram.

This investigation was performed in accordance with relevant guidelines/regulations (the Declaration of Helsinki) and approved by the University of Texas MD Anderson Cancer Center (UTMDACC) Institutional Review Board. Potentially eligible patients were identified by providers in the gastrointestinal or diagnostic imaging clinics at UTMDACC in a consecutive manner from December 2014 to September 2015. Once potentially eligible patients were identified, the provider contacted the study team to approach the patient in clinic. A total of 135 gastrointestinal and pancreatic cancer survivors were approached during their regularly scheduled visit by the study team. One hundred and three patients signed the informed consent document, underwent their routine CT scan for medical purposes, and then completed one whole body DXA scan within 48 hours of their CT scan, in the Behavioral Research and Treatment Center at UTMDACC. In addition to the routine CT scan and DXA scan, participants then completed patient demographic and cancer history questionnaires. A total of 99 pairs of DXA and CT scan measures were used for VAT area analysis. One of the participants’ data were removed from the analyses due to an unreadable CT scan resulting in the inability to determine VAT area. A total of 88 pairs of DXA and CT scan measures were used for VAT volume analysis; 12 CT scans did not include the full region of interest required for CT VAT volume analysis (Fig. 5).

### Demographics & cancer history

Participants were provided with standard questionnaires regarding demographic information and cancer history.

### Computed tomography

The General Electric Lightspeed CT scanner (GE Medical Systems, Milwaukee, WI) was used to capture visceral adiposity and images were saved as Digital Imaging and Communications in Medicine (DICOM) files for analysis by a physician. Standard procedures were followed, using 120 kV, 2.5 mm thickness, and a field of view of 50 cm. The average radiation dose exposure of the full abdominal and pelvis scan with contrast is approximately 16 mSv^29^.

VAT area was estimated at L_4_-L_5_ intervertebral space with free-form segmentation that closely approximated the Alberta protocol, a standard attenuation-constrained segmentation protocol, via the built-in tools provided by the Slice-O-Matic software (TomoVision, Canada). The commercially available Slice-O-Matic software has previously been shown as an accurate method to measure VAT area with an average coefficient of variation of 0.2–3.4%^30,31^, and is an established validated (i.e. demonstrated repeatability and stability) method for measuring VAT area^31,32^.

VAT volume was estimated with newly developed in-house software, the Volumetric Quantification of Visceral Adipose Tissue using CT^21^. The calculation method has been validated using a tissue phantom, and the mean intra- and inter-observer coefficients of variation were 5.9% and 8.5%, respectively^21^. Under this approach, a full CT abdomen and pelvis scan was conducted and the image was imported into the software program. Here, the region of interest was defined from the dome of the liver to the tip of the femoral heads. The abdominal contents were then defined with a series of ellipses throughout the region of interest, thereby separating visceral and subcutaneous adipose tissues. The slice thickness and pixel number were used to quantify VAT volume, as is the standard of practice.

### Dual energy X-ray absorptiometry

VAT area and volume was estimated by whole body DXA scan via the Hologic Discovery A series equipped with APEX version 4.0 software (Bedford, MA) following standard procedures. The average radiation dose exposure of a whole-body DXA scan for DXA systems is approximately 0.1–4.9 µSV^20^. The DXA software estimates VAT area from an automatically marked region of interest at L_4_-L_5_ and estimates VAT volume based on the VAT area estimation, slice thickness, and a general scaling factor^18^. Prior to testing, quality control calibration procedures for whole body DXA scan was performed following manufacturer’s guidelines and the average coefficient of variation for was 0.273%. Whole-body DXA has been previously validated for VAT measurement^20,22^.

### Statistical analysis

Data were analyzed with SPSS statistical software package, version 23 (Chicago, IL). Descriptive statistics were computed for participant characteristics and demographic data. Two-sample t-tests were used to determine differences in participant characteristics by sex. The Bland-Altman method was used to determine the level of agreement between CT scan and DXA measures for the total sample and by sex^33^. In this method the difference between each participant’s CT and DXA VAT measures (CT VAT minus DXA VAT) was plotted against the average of each participant’s CT and DXA VAT measures (the sum of CT and DXA VAT measures divided by two). Further, the bias, which is the average of the difference between paired measures, was plotted along with the “limits of agreement,” defined as the 95% confidence intervals of the mean difference between paired measures. As secondary analyses, we conducted a simple linear regression with the CT scan and DXA measures being the dependent and independent variables, respectively, to estimate both the correlation coefficient between the two measures and the residual standard deviation estimate for the total sample and by sex. The residual standard deviation estimate was regarded as a measurement of accuracy of the underlying regression line used for predicting the CT scan measure based on the DXA measure^23^.

## Data Availability

The dataset generated and analyzed during the current study are available from the corresponding author on reasonable request.
